# Welding Fume Instillation in Isolated Perfused Mouse Lungs—Effects of Zinc- and Copper-Containing Welding Fumes

**DOI:** 10.3390/ijms23169052

**Published:** 2022-08-12

**Authors:** Julia Krabbe, Thomas Kraus, Hanif Krabbe, Christian Martin, Patrick Ziegler

**Affiliations:** 1 Institute of Occupational, Social and Environmental Medicine, Medical Faculty, RWTH Aachen University, Pauwelsstraße 30, 52074 Aachen, Germany; 2 European Vascular Centre Aachen-Maastricht, Department of Vascular Surgery, Medical Faculty, University Hospital RWTH Aachen, Pauwelsstraße 30, 52074 Aachen, Germany; 3 Institute of Pharmacology and Toxicology, Medical Faculty, RWTH Aachen University, Wendlingweg 2, 52074 Aachen, Germany

**Keywords:** isolated perfused lung, welding fumes, zinc, copper, myeloperoxidase

## Abstract

Zinc- and copper-containing welding fumes can cause systemic inflammation after exposure in humans. Recent ex vivo studies have shown that the observed inflammation originates from exposed immune cells. In vitro studies identified the soluble fraction of metal particles as the main effectors. Isolated perfused mouse lungs (IPLs) were perfused and ventilated for 270 min. Lungs were instilled with saline solution (control), welding fume particle suspension (WFs) or the soluble fraction of the welding fumes (SF-WFs). Bronchoalveolar lavage fluid (BALF) and perfusate samples were analyzed for cytokine levels and lung tissue mRNA expression levels were analyzed via RT-PCR. All lungs instilled with WFs did not complete the experiments due to a fatal reduction in tidal volume. Accordingly, IL-6 and MPO levels were significantly higher in BALF of WF lungs compared to the control. IL-6 and MPO mRNA expression levels were also increased for WFs. Lungs instilled with SF-WFs only showed mild reactions in tidal volume, with BALF and mRNA expression levels not significantly differing from the control. Zinc- and copper-containing welding fume particles adversely affect IPLs when instilled, as evidenced by the fatal loss in tidal volume and increased cytokine expression and secretion. The effects are mainly caused by the particles, not by the soluble fraction.

## 1. Introduction

Welding fumes are airborne metal particles produced by a welding process under high heat. The composition and size of the particles depend on the selected welding process, as well as on the base and filler materials. Typically, they have a particle size between 1000 and 50 nm [1,2]. This allows them to penetrate the alveoli, where they are deposited during inhalation.

Zinc- and copper-containing welding fumes can cause systemic inflammation in humans after exposure [3]. This condition known as metal fume fever or ‘Monday morning fever’ usually does not require specific therapy and occurs frequently in welders [4,5,6]. The inflammatory reaction has been described both after exposure to zinc- and copper-containing welding fumes, and after exposure to only zinc- or only copper-containing welding fumes [7]. Contrary to symptoms, which do not recur with repeated exposure, inflammation persists and poses a potential risk to welders [3]. Interestingly, common symptoms include myalgia, headache and fever, which is primarily indicative of systemic inflammation. Although exposure is by inhalation, respiratory- or lung-related symptoms, e.g., cough, are less common [8,9].

Recent ex vivo studies revealed that the observed inflammation does not originate from the lung cells themselves [10], but rather stems from immune cells in the blood reacting to the welding fume particles [11]. The lungs would therefore be an entry point rather than a site of inflammation. However, in these ex vivo studies, exposure to welding fume particles was via the incubation of suspended particles in incubation buffers. In addition, the model of lung slices did not offer the possibility of observation under ventilation. Therefore, a transfer to a realistic inhalation exposure would be far-fetched.

In an interesting in vitro study in A549 lung cells, the soluble fraction of welding fumes was identified as the main effector via an oxidative stress mechanism [12]. In another study in human bronchial epithelial cells, it was found that welding fumes with higher solubility are more toxic [13].

In this study, we investigated the effects of welding fume particles instilled into isolated perfused mouse lungs (IPLs) to characterize the exerted effects in a model with ventilation and pulmonary perfusion. To identify possible effects due to the solubility of the welding fumes tested, instillation was also performed with the soluble fraction of welding fumes. This way, particle-associated effects could also be identified. The response of the respiratory tract and vasotonus was recorded, as well as the inflammatory response at the protein and gene level.

## 2. Results

### 2.1. IPL—Experimental Results

#### 2.1.1. Success Rate of IPLs

**Figure 1:**
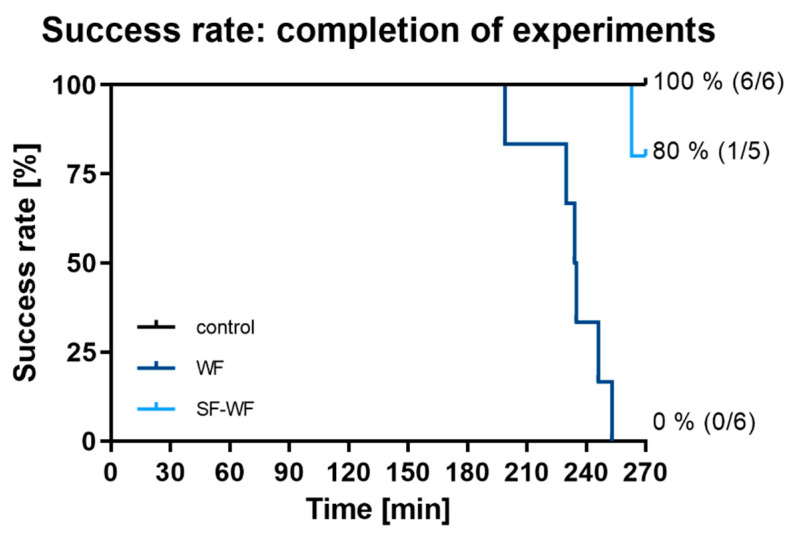
**Influence of welding fume instillation on IPLs.** Success rates of groups over 270 min of experiment. Control and WF n = 6, SF-WF n = 5.

In the control group (n = 6), all IPLs remained stable throughout the 270 min of the experiment (Figure 1—black). In the group instilled with welding fumes (WF, n = 6), all lungs dropped out between minute 199 and 253 of the experiment (Figure 1—dark blue). Instillation with the soluble fraction of the welding fumes (SF-WF, n = 5) resulted in one failure at minute 263, resulting in a success rate of 80% (Figure 1—light blue).

#### 2.1.2. Tidal Volume, Airway Resistance and Perfusion Pressure

**Figure 2:**
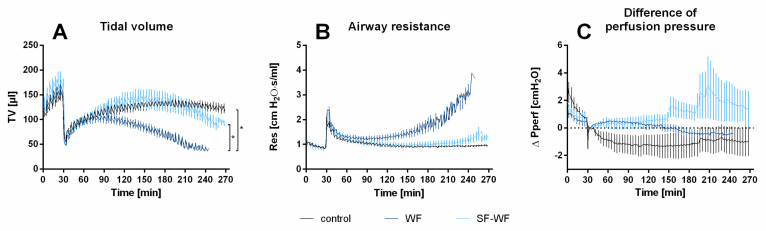
**Influence of welding fume instillation on tidal volume, airway resistance and perfusion pressure.** (**A**)**:** Tidal volume. (mean ± SEM); (**B**): Airway resistance (mean ± SEM); (**C**): Difference in perfusion pressure. The difference Δ µL is normalized to the initial value at 30 min. (mean ± SEM); * = *p* < 0.05, n = 6 per group except WF-SF (n = 5). The statistical analysis was conducted for the interval of 0 to 210 min.

After a 30 min baseline of perfusion and ventilation, the lungs of the mice were instilled with saline solution (control), welding fume particle suspension (WFs) or the soluble fraction of welding fumes (SF-WFs), resulting in an immediate decrease in tidal volume (Figure 2A). This was followed by a partial recovery of tidal volume over time. Lungs instilled with WFs experienced a significant decrease in tidal volume over time (Figure 2A). Although there tended to be higher airway resistance in lungs instilled with WFs during the first 210 min of the experiment, there were no statistically significant differences between all three groups (Figure 2B). No significant differences were observed in the differences in perfusion pressure for all groups (Figure 2C).

#### 2.1.3. Cytokine Levels in BALF and Perfusate

**Figure 3:**
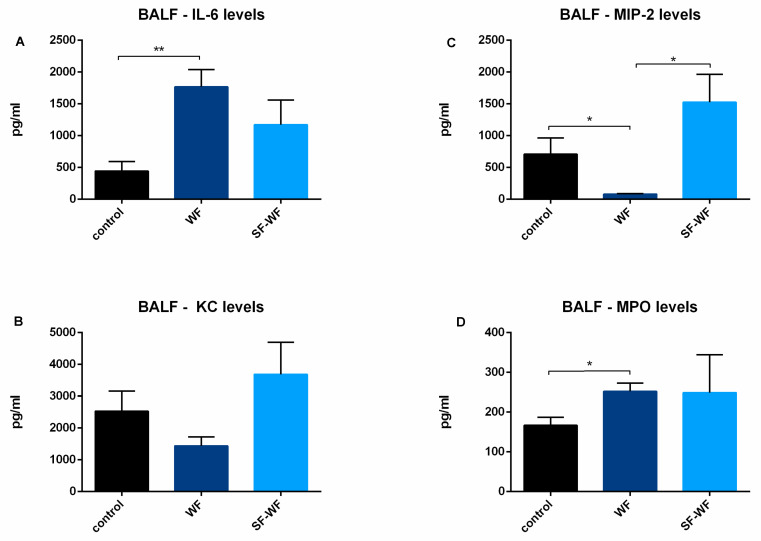
**Influence of welding fume instillation: Cytokine release of isolated perfused lungs into BALF.** (**A**): IL-6 levels in BALF (mean ± SEM), (**B**): KC levels in BALF (mean ± SEM), (**C**): MIP-2 levels in BALF (mean ± SEM), (**D**): MPO levels in BALF (mean ± SEM.). * = *p* < 0.05, ** = *p* < 0.01, control and WF n = 6, SF-WF n = 5.

Cytokine levels of IL-6 were significantly increased in lungs instilled with welding fumes compared to the control lungs (Figure 3A). Interestingly, the opposite was observed for MIP-2 (Figure 3B). Here, the control lungs and the lungs instilled with the soluble fraction of the welding fumes (SF-WFs) had significantly higher levels than the lungs instilled with welding fumes (WFs). No differences were found in KC levels (Figure 3C). For MPO levels, significantly lower levels were found in the control lungs compared to the WFs (Figure 3D).

**Figure 4:**
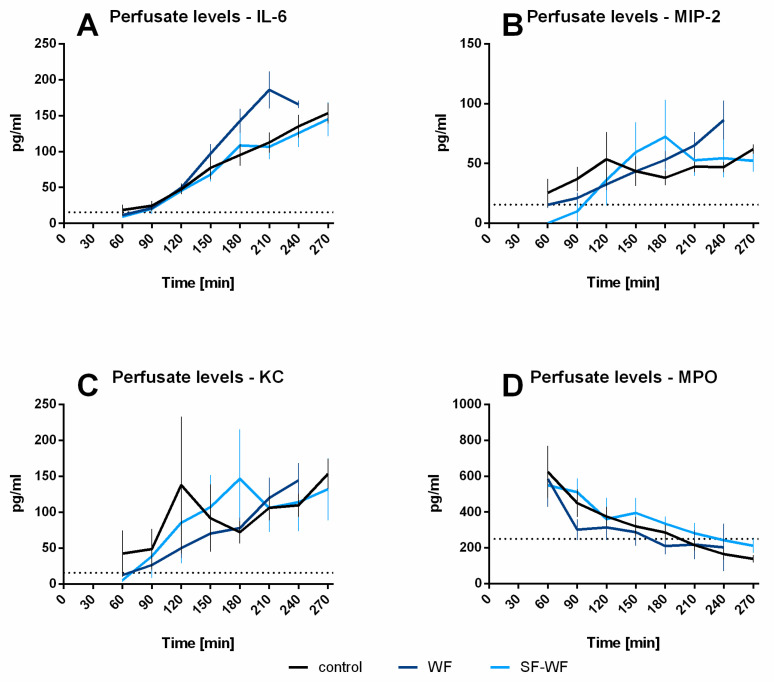
**Influence of welding fume instillation: Cytokine release of isolated perfused lungs into perfusate.** (**A**): IL-6 levels in perfusate (mean ± SEM), (**B**): KC levels in perfusate (mean ± SEM), (**C**): MIP-2 levels in perfusate (mean ± SEM), (**D**): MPO levels in perfusate (mean ± SEM), control and WF n = 6, SF-WF n = 5.

Although a tendency towards higher IL-6 levels was observed in the WF-instilled lungs, no statistically significant differences occurred in the IL-6 levels in the perfusate (Figure 4A). Similarly, no differences could be observed for MIP-2 (Figure 4B), KC (Figure 4C) and MPO (Figure 4D).

#### 2.1.4. mRNA Expression Levels

**Figure 5:**
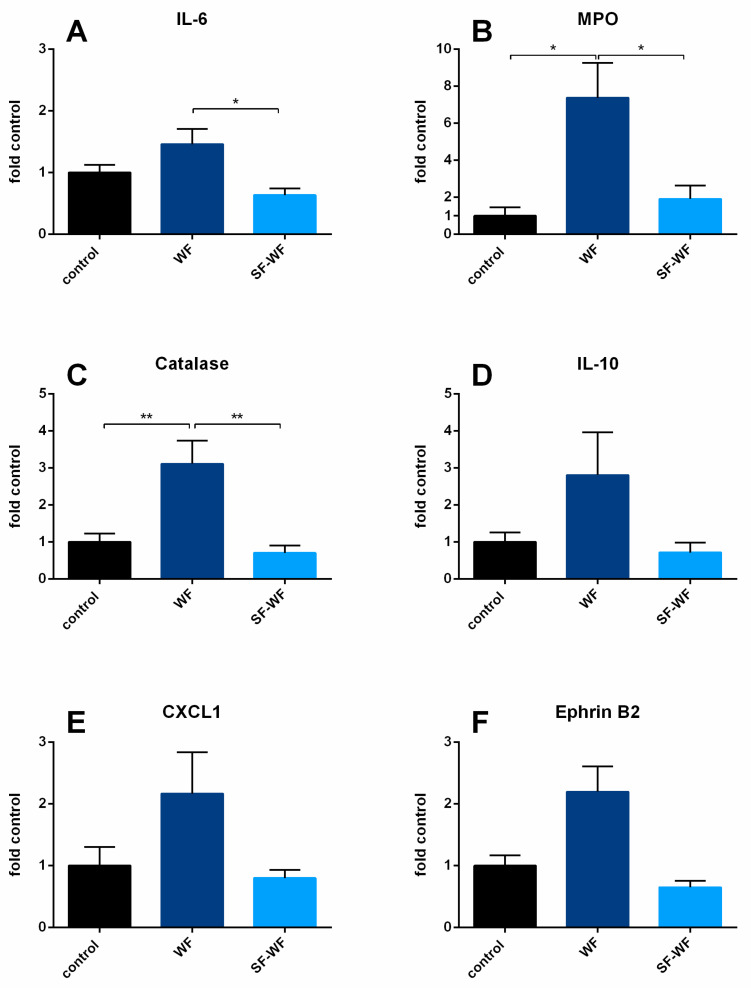
**Influence of welding fume instillation: mRNA expression of isolated perfused lungs.** (**A**): IL-6 mRNA expression levels (mean ± SEM), (**B**): MPO mRNA expression levels (mean ± SEM), (**C**): catalase mRNA expression levels (mean ± SEM), (**D**): IL-10 mRNA expression levels (mean ± SEM), (**E**): CXCL-1 mRNA expression levels (mean ± SEM), (**F**): EphrinB2 mRNA expression levels (mean ± SEM), control and WF n = 6, SF-WF n = 5. * = *p* < 0.05, ** = *p* < 0.01.

Similar to BALF, a significant increase in mRNA expression for IL-6 (Figure 5A) and MPO (Figure 5B) was detected in lungs instilled with welding fume particles (WFs) compared to the lungs instilled with the soluble fraction of the welding fumes (SF-WFs). In addition, MPO mRNA expression was higher compared to the control lungs (Figure 5B). Catalase expression increased in the WF lungs (Figure 5C). Although there was a tendency for higher expression for IL-10 (Figure 5D), CXCL-1 (Figure 5E) and EphrinB2 (Figure 5F) in WF lungs, no significant differences were observed. For the mRNA expression of Ephrin type-B receptor 4 (EphB4), C-X-C motif chemokine 2 (CXCL2), IL-1β, TNFα, protein-tyrosine phosphatase 1B (PTP1B) and matrix metalloproteinase-7 (MMP-7) (see Supplementary Materials, Figure S1), no altered induction was detected either.

## 3. Discussion

The present study demonstrates the effect of zinc- and copper-containing welding fumes instilled into isolated perfused mouse lungs. All lungs instilled with welding fume particles terminated the experiments with a decrease in tidal volume. BALF of these lungs showed significantly increased IL-6 and MPO levels compared to the controls, indicating the inflammation of the lungs by both inflammatory mediators and cells. This was also demonstrated for mRNA expression in lung tissue for IL-6, MPO and catalase. Lungs instilled with the soluble fraction of the welding fumes only showed certain tendencies, but no pronounced signs of inflammation.

Intratracheal instillation of welding fume particles is a sufficient and reliable exposure technique in ex vivo experiments. In the present study, welding fume particles suspended in sodium chloride were instilled. Instillation of 50 µL of liquid into the IPLs of mice usually results in a sudden drop in tidal volume which recovers over the next few hours, as shown in the control lungs of this study. Surprisingly, the lungs instilled with welding fume particles showed a decrease and failure after 180 min and later, while the lungs instilled with the soluble fraction only showed a decrease in tidal volume with a single failure towards the end of the experiments. This suggests an effect of welding fume particles in addition to the already dissolved zinc and copper ions in the soluble fraction. In studies investigating the toxicity of metal particles in vitro, the soluble fraction was identified as the main effector of oxidative stress [12], and in human bronchial epithelial cells, welding fume particles with higher solubility were observed to be more toxic [13]. In contrast, IPL lungs were significantly more damaged by welding fume particles than by their soluble fraction as detected by increased cytokine and MPO release. This could be due to multiple effects. First, the deposition of welding fume particles in the lungs with consecutive dissolution of zinc and copper ions: welding fume particles are deposited in the alveoli and they are mainly in lung mucus after instillation. In vivo, the particles are phagocytized by scavenger cells and redeposited in their phagolysosomes [14] with consecutive solution in an acidic environment [15,16]. Zinc- and copper-containing welding fumes are known to inhibit the protein-tyrosine phosphatase 1B (PTP1B), resulting in an increased secretion of cytokines, e.g., IL-6 [11].

This main mechanism of welding fume action is only of secondary importance in IPLs, as only a few residential phagocytes are present in the lungs during the experiments. Migration to the lungs is not possible in the IPL setup. Therefore, the dissolution of zinc and copper ions in lung mucus could be the main effect. Zinc- and copper-containing welding fumes are partially soluble in pH neutral fluids [10] and mucus has an approximately neutral pH (pH 7.2–7.3) [17,18]. Deposited welding fume particles could represent a kind of depot in the mucus which dissolves over time and enhances possible zinc and copper ion effects.

In addition, the mechanisms of lung clearance and the effects of particles on it are observed in ventilated lungs. The instillation of particles, in contrast to the application of soluble fraction, could lead to an overload of the lung clearance system with consecutive lung failure. A possible constricting or obstructing effect of the particles is rather unlikely since zinc- and copper-containing welding fume particles used in these experiments have a diameter of about 120 nm [10] and do not accumulate to visible compounds. Furthermore, there would have been a sudden increase in airway resistance, which was not detected, but a steady increase over time was seen.

The pattern of increases in tidal volume and airway resistance of lungs instilled with welding fume particles compared to lungs instilled with control and soluble fraction is in accordance with the observed inflammatory reaction in the lungs. Both mediator and cell-based lung inflammation were indicated by increased IL-6 and MPO levels in the BALF. This suggests an induction of inflammation in the lung cells as well as in the resident scavenger cells, most likely neutrophil granulocytes as indicated by increased MPO levels. This observed inflammatory response after lung exposure to welding fumes is in accordance with studies examining lung histology and BALF in welders and rats. In these, considerable cell influx into the lungs and increased neutrophil concentrations in the BALF was observed [19,20]. Increased IL-6 and IL-8 levels were observed in the BALF of volunteers exposed to zinc oxide [21]. The increase in MPO in the present study indicates that neutrophil granulocytes are still present in lung tissue. At the same time, neutrophils are no longer present in the pulmonary vasculature, as indicated by the decreasing MPO levels in perfusate. In contrast, IL-6 levels increased over time with a tendency of higher IL-6 levels in WF lungs in accordance with higher IL-6 levels in the BALF. This could mark the first phase of welding fume fever, whereby inflammation is not limited to the lungs, but elevated IL-6 levels in the blood also trigger increased production and secretion of C-reactive protein (CRP) in the liver and initiate an acute phase response [22]. Those are previously documented steps in the occurrence of welding fume fever in volunteers exposed to zinc- and copper-containing welding fumes [1,5], indicating the same underlying mechanisms and processes as in humans exposed to welding fumes. Interestingly, a certain difference in systemic inflammatory reaction to zinc- and copper-containing welding fumes could be observed in past studies with some subjects not experiencing CRP level increases at all [23,24]. An individual susceptibility was postulated although no potential mechanism was described. Similar to approaching cancer therapy with personalized diagnostics and therapy [25], welding fume effects should be investigated regarding individual susceptibility. However, this concept could not be investigated in this study since the laboratory mice used were all obtained from one manufacturer at a narrow time frame and should have been genetically identical. Future inhalation studies with human subjects should address the potential individual susceptibility and their role in welding fume effects.

Studies with human volunteers or welders reported increased levels of IL-6, IL-8 and TNFalpha in BALF [21]. In ex vivo experiments with precision-cut lung slices from humans and animals, no pronounced inflammation could be detected [10]. In the present study, the mRNA expression of IL-6, MPO and catalase was significantly increased for lungs affected by welding fume particles (WFs), while only trends were observed for IL-10, CXCL-1 and EphrinB2. Together with the increased levels of IL-6 and MPO in BALF, a clear inflammatory reaction of the lungs themselves could be detected. Thus, the lungs seem to be more than just the entry point for the effects exerted by welding fume particles, but rather the origin of the inflammation with consecutive spread to the vascular system, which develops into a systemic inflammation known as metal fume fever. Interestingly, although zinc- and copper-containing welding fumes were shown to inhibit PTP1B, no alteration of mRNA of PTP1B could be detected. It is possible that the time course of experiments was too short to detect effects on the mRNA expression of PTP1B. EphrinB2 and EphB4 as ligand and consecutive receptors also showed no significant change in expression although ephB4 has been shown to indicate lung injury in mouse IPLs and in vivo [26]. Differences in those two studies could be due to different infliction of lung injury. In the present study, particle-related effects led to a sudden drop in tidal volume, while in the other study, overventilation with an increased tidal volume led to lung injury [26].

Rapid induction of gene expression in lung tissue has also been observed for other inflammatory agents such as cigarette smoke [27]. Here, KC, MMP-9 and MMP-12, but not IL-6 or MIP-2 expression, were increased in mice after cigarette smoke inhalation. However, cigarette smoking is described to exert main effects via IL-1 receptor/myeloid differentiation primary response 88 (MyD88) signaling and IL-1β secretion [28,29,30], and in this study, no relevant IL-1β mRNA expression could be found. Thus, effects exerted by different smokes depend on smoke composition and consecutively differ in molecular signaling.

In addition to increased protein and mRNA expression levels indicating inflammation, oxidative stress was also observed in IPLs caused by zinc- and copper-containing welding fumes. MPO is one of the major enzymes involved in the generation of reactive oxygen species (ROS) by catalyzing the conversion of hydrogen peroxide into ROS [31]. Catalase is a hydrogen peroxide-dismutant enzyme that exerts antioxidant effects [32]. Both have shown elevated mRNA expression levels indicative of oxidative stress from zinc-and copper-containing welding fumes after contact with the lungs. MPO was also detectable in BALF in IPLs and in blood samples from human subjects exposed to the same welding fumes in this study [33]. MPO is also a prognostic marker for cardiovascular risks and events [31]. Interestingly, MPO levels correlated with CRP levels and neutrophil granulocytes in subjects exposed to zinc- and copper-containing welding fumes [24], making it a promising candidate for monitoring welding fume-induced inflammation.

In summary, this study was able to show that zinc- and copper-containing welding fumes induce both inflammatory and oxidative stress in IPLs. Since the main effects were caused by instilled particles and not their soluble fraction, further studies should clarify whether other particle effects should be considered. Myeloperoxidase (MPO) again proved to be a reliable candidate for a marker indicating inflammation in the lungs caused by zinc- and copper-containing welding fumes and should be further investigated in future human studies.

## 4. Materials and Methods

### 4.1. Animals

All animal care and experimental procedures were performed according to the rules of the Directive 2010/63/EU of the European Parliament. They were approved by the Institute for Laboratory Animal Science and Experimental Surgery, Medical Faculty, RWTH Aachen University (approval-ID: 50053A4).

Male C57BL/6Ns (20 ± 3 g) obtained from Janvier (Le Genest-Saint-Isle, France) were used as lung donors for IPLs. They were randomly assigned to one of three groups: instillation with 0.9% sodium chloride (control), welding fume particles (WFs) or the soluble fraction of WFs (SF-WFs). All mice were housed in an individually ventilated cage system (Positive/Negative Control IVC; Allentown Inc., Allentown, PA, USA) according to the recommendations of the Federation of Laboratory Animal Science Associations (FELASA) and German Society of Laboratory Animal Science (GV-SOLAS).

### 4.2. Agents

Gelafundin 4% was obtained from B. Braun (Melsungen, Germany). The buffer supplements were all obtained from Merck (Darmstadt, Germany), except sodium chloride, potassium chloride, D-(+)-glucose monohydrate, calcium nitrate tetrahydrate, MEM amino acids (50×), MEM non-essential amino acids (100×) and MEM vitamins (100×), which were from Sigma-Aldrich (Steinheim, Germany), and glutathione, Dulbecco’s phosphate-buffered saline (DPBS) and ultraglutamine which were purchased from Lonza (Basel, Switzerland). Pentobarbital (Narcoren) was purchased from Merial (Hallbergmoos, Germany).

### 4.3. Welding Fume Particle Production and Characterization

The production and characterization of the welding fume particle has been described before [17]. In brief, the welding fumes were produced using metal-inert-gas (MIG) brazing of hot-dip-galvanized steel using a low alloy copper wire and an impulse arc process. Welding was performed under a sampling hood; the fumes were collected on an ashless paper filter and the particles were removed using a small brush. The median particle diameter was 120 nm. Particles were polydisperse with a geometric standard deviation of 1.6. The welding fume samples used in this study contained 53% zinc and 24% copper using Atomic Absorption Spectrometry techniques [17].

### 4.4. Isolated Perfused Mouse Lung Preparation (IPL)

**Figure 6:**
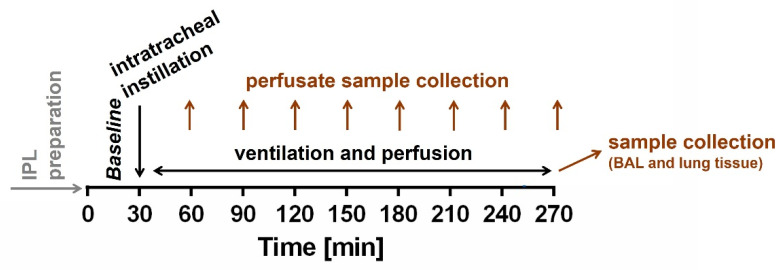
**Scheme of experiments:** IPLs were prepared from mice and ventilated and perfused for the next 30 min as baseline. Afterwards, the lungs were instilled via tracheal cannula with a 0.9% sodium chloride (control), welding fume particles (WFs) or the soluble fraction of WFs (SF-WFs). The experiments were planned for 270 min total. After 60 min, perfusate sample collection began and was repeated every 30 min. At the end of the experiments, BAL and lung tissue were collected.

IPLs were prepared from mice as described before [27,34] in the IPL-1 system (Hugo Sachs Elektronik, March Hugstetten, Germany) and ventilated with a respiratory rate of 90 breaths per minute and an end-inspiratory pressure of −8 cm H_2_O and an end-expiratory pressure of −3 cm H_2_O, resulting in a tidal volume of ~250 µL, for the next 30 min as baseline (Figure 6). Afterwards, mice lungs were instilled via tracheal cannula with a volume of 50 µL of fluid containing 0.9% sodium chloride (control), 200 µg of welding fume particles in 50 µL (WFs) or the soluble fraction of WFs (SF-WFs). The soluble fraction was obtained by incubation of welding fume particles in sodium chloride at 37 °C and centrifugation afterwards. Non-recirculating perfusion via pulmonary artery and left ventricle was performed at 0.5 mL/min resulting in a pulmonary inflow perfusion pressure of 0.5 up to 5 cm H_2_O. The experiments were planned for a ventilation and perfusion of 240 min after instillation. Thus, the experiments were conducted for 270 min. The perfusing buffer contained polysuccinated bovine gelatin and was added with various supplements including sodium phosphate and sodium hydrogen carbonate as described before [34]. All data were transmitted to a computer and analyzed by the Pulmodyn software (Hugo Sachs Elektronik, March Hugstetten, Germany).

### 4.5. Enzyme-Linked Immunosorbent Assays (ELISA)

Perfusate samples, as well as BALF were analyzed for cytokine levels. The perfusate samples were collected after 60, 120, 180, 210, 240 and 270 min of perfusion. BALF was collected from the right lung by lavage with 500 µL of phosphate-buffered saline (PBS) immediately after the end of ventilation. Levels of Interleukin 6 (IL-6), Keratinocyte-Derived Chemokine (KC), macrophage inflammatory protein (MIP-2), tumor necrosis factor alpha (TNFα) and myeloperoxidase (MPO) were determined in perfusate samples and BALF. The concentrations were measured by a commercially available enzyme-linked immunosorbent assay (ELISA) kit with specific monoclonal antibodies according to the manufacturer’s instructions (R&D Systems, Inc., Minneapolis, MN, USA).

### 4.6. Reverse Transcription Quantitative Polymerase Chain Reaction (RT-qPCR)

After the experiments, the left lung was snap frozen in liquid nitrogen and stored at −80 °C. Samples were then ground with a mortar over liquid nitrogen for RNA extraction. The homogenized solution was purified using a Qiagen shredder column and total RNA was extracted with the RNeasy Mini Kit. cDNA was synthesized using random hexamers primer and Superscript III reverse transcriptase according to the manufacturer’s instructions (Invitrogen, Carlsbad, CA, USA). Equal amounts of total RNA from various samples were used for RT-PCR reactions. The final cDNA was then subjected to real-time PCR (StepOnePlus Real-Time PCR Systems, Applied Biosystems) using TaqMan Arrays predesigned 96-well plates without technical replicates. The following genes were amplified: Ptpn1 (assay ID: Mm00448427_m1); Efnb2 (assay ID: Mm00438670_m1); Ephb4 (assay ID: Mm01201157_m1); Mpo (assay ID: Mm00447885_m1); Il6 (assay ID: Mm00446190_m1); Cxcl2 (assay ID: Mm00436450_m1); Tnf (assay ID: Mm00443258_m1); Il10 (assay ID: Mm01288386_m1); IL-1b (assay ID: Mm00434228_m1); and Cat (assay ID Mm00437992_m1). The reference gene was determined among a set of four candidates: 18s rRNA (assay ID: Hs99999901_s1); GapdH (assay ID: Mm01197698_m1); GusB (assay ID: Mm01197698_m1); and RPLP0 (assay IDMm00725448_s1). GapdH was identified as the most stable gene in our assay. Relative quantification (RQ) of gene expression was determined using the 2^−ΔΔCt^ method.

### 4.7. Statistics

Data analysis was performed using SAS 9.4 (SAS Institute Inc., Cary, NC, USA). All data are shown as mean ± SEM and n indicates the number of animals. Analysis was carried out using general linear mixed model analysis (Proc Glimmix) assuming a normal distribution. Additionally, data were assumed to be derived from lognormal (cytokines) or beta (percentage data) distribution and residual plots and the Shapiro–Wilk test were used as diagnostics. In the case of heteroscedasticity, the degrees of freedom were adjusted by the Kenward–Roger approximation. *p* values were always adjusted by the simulated-Shaffer procedure. Regarding repeated measurements (Figure 2 and Figure 4), data were reduced to measurements of every 30 min. Since dropouts occurred between minute 199 and 263 of the experiments and only one lung dropped out before 210 min, analysis was conducted for the interval between 0 and 210 min for tidal volume, airway resistance and perfusion pressure (Figure 2), as well as for perfusate cytokine levels (Figure 4) to include most lungs over the time course of the experiments.

Data were plotted with GraphPad Prism 6 (GraphPad, La Jolla, CA, USA). RT-PCR data were normalized to the mean of the control group for every assessed gene. Analysis differences were assumed to be significant with *p* < 0.05.

## Data Availability

The data presented in this study are available on request from the corresponding author.

## Supplementary Materials

The following supporting information can be downloaded at: https://www.mdpi.com/article/10.3390/ijms23169052/s1.

## References

[1] Brand P., Lenz K., Reisgen U., Kraus T. (2013). Number Size Distribution of Fine and Ultrafine Fume Particles from Various Welding Processes. Ann. Occup. Hyg..

[2] Berger F., Bernatíková Š., Kocůrková L., Přichystalová R., Schreiberová L. (2021). Occupational Exposure to Nanoparticles Originating from Welding—Case Studies from the Czech Republic. Med. Pr..

[3] Krabbe J., Beilmann V., Gerhards B., Markert A., Thomas K., Kraus T., Brand P. (2019). The Effects of Repeated Exposure to Zinc- and Copper-Containing Welding Fumes on Healthy Volunteers. J. Occup. Environ. Med..

[4] Kaye P., Young H., O’Sullivan I. (2002). Metal Fume Fever: A Case Report and Review of the Literature. Emerg. Med. J..

[5] Wong A., Greene S., Robinson J. (2012). Metal Fume Fever—A Case Review of Calls Made to the Victorian Poisons Information Centre. Aust. Fam. Physician.

[6] Greenberg M.I., Vearrier D. (2015). Metal Fume Fever and Polymer Fume Fever. Clin. Toxicol. Phila. Pa.

[7] Markert A., Baumann R., Gerhards B., Gube M., Kossack V., Kraus T., Brand P. (2016). Single and Combined Exposure to Zinc- and Copper-Containing Welding Fumes Lead to Asymptomatic Systemic Inflammation. J. Occup. Environ. Med..

[8] El-Zein M., Infante-Rivard C., Malo J., Gautrin D. (2005). Is Metal Fume Fever a Determinant of Welding Related Respiratory Symptoms and/or Increased Bronchial Responsiveness? A Longitudinal Study. Occup. Environ. Med..

[9] El-Zein M., Malo J.-L., Infante-Rivard C., Gautrin D. (2003). Prevalence and Association of Welding Related Systemic and Respiratory Symptoms in Welders. Occup. Environ. Med..

[10] Krabbe J., Esser A., Kanzler S., Braunschweig T., Kintsler S., Spillner J., Schröder T., Kalverkamp S., Balakirski G., Gerhards B. (2018). The Effects of Zinc- and Copper-Containing Welding Fumes on Murine, Rat and Human Precision-Cut Lung Slices. J. Trace Elem. Med. Biol..

[11] Bleidorn J., Alamzad-Krabbe H., Gerhards B., Kraus T., Brand P., Krabbe J., Martin C. (2019). The Pro-Inflammatory Stimulus of Zinc- and Copper-Containing Welding Fumes in Whole Blood Assay via Protein Tyrosine Phosphatase 1B Inhibition. Sci. Rep..

[12] McNeilly J.D., Heal M.R., Beverland I.J., Howe A., Gibson M.D., Hibbs L.R., MacNee W., Donaldson K. (2004). Soluble Transition Metals Cause the Pro-Inflammatory Effects of Welding Fumes in Vitro. Toxicol. Appl. Pharmacol..

[13] McCarrick S., Wei Z., Moelijker N., Derr R., Persson K.-A., Hendriks G., Odnevall Wallinder I., Hedberg Y., Karlsson H.L. (2019). High Variability in Toxicity of Welding Fume Nanoparticles from Stainless Steel in Lung Cells and Reporter Cell Lines: The Role of Particle Reactivity and Solubility. Nanotoxicology.

[14] Xia T., Kovochich M., Liong M., Mädler L., Gilbert B., Shi H., Yeh J.I., Zink J.I., Nel A.E. (2008). Comparison of the Mechanism of Toxicity of Zinc Oxide and Cerium Oxide Nanoparticles Based on Dissolution and Oxidative Stress Properties. ACS Nano.

[15] Cho W.-S., Duffin R., Howie S.E., Scotton C.J., Wallace W.A., MacNee W., Bradley M., Megson I.L., Donaldson K. (2011). Progressive Severe Lung Injury by Zinc Oxide Nanoparticles; the Role of Zn2+ Dissolution inside Lysosomes. Part. Fibre Toxicol..

[16] Studer A.M., Limbach L.K., Van Duc L., Krumeich F., Athanassiou E.K., Gerber L.C., Moch H., Stark W.J. (2010). Nanoparticle Cytotoxicity Depends on Intracellular Solubility: Comparison of Stabilized Copper Metal and Degradable Copper Oxide Nanoparticles. Toxicol. Lett..

[17] Pezzulo A.A., Tang X.X., Hoegger M.J., Abou Alaiwa M.H., Ramachandran S., Moninger T.O., Karp P.H., Wohlford-Lenane C.L., Haagsman H.P., van Eijk M. (2012). Reduced Airway Surface PH Impairs Bacterial Killing in the Porcine Cystic Fibrosis Lung. Nature.

[18] Jayaraman S., Joo N.S., Reitz B., Wine J.J., Verkman A.S. (2001). Submucosal Gland Secretions in Airways from Cystic Fibrosis Patients Have Normal [Na(+)] and PH but Elevated Viscosity. Proc. Natl. Acad. Sci. USA.

[19] Antonini J.M., Roberts J.R., Schwegler-Berry D., Mercer R.R. (2013). Comparative Microscopic Study of Human and Rat Lungs After Overexposure to Welding Fume. Ann. Occup. Hyg..

[20] Halatek T., Stanislawska M., Kaminska I., Cieslak M., Swiercz R., Wasowicz W. (2017). The Time-Dependent Health and Biochemical Effects in Rats Exposed to Stainless Steel Welding Dust and Its Soluble Form. J. Environ. Sci. Health Part A.

[21] Blanc P.D., Boushey H.A., Wong H., Wintermeyer S.F., Bernstein M.S. (1993). Cytokines in Metal Fume Fever. Am. Rev. Respir. Dis..

[22] Tanaka T., Kishimoto T. (2014). The Biology and Medical Implications of Interleukin-6. Cancer Immunol. Res..

[23] Brand P., Beilmann V., Krichel T., Merizian J., Schmidt K., Kraus T., Krabbe J. (2020). No Observed Effect Level (NOEL) for Systemic Inflammation by Copper and Zinc in Welding Fumes. J. Occup. Environ. Med..

[24] Brand P., Beilmann V., Thomas K., Kraus T., Krichel T., Reisgen M., Schmidt K., Krabbe J. (2019). The Effects of Exposure Time on Systemic Inflammation in Subjects With Exposure to Zinc- and Copper-Containing Brazing Fumes. J. Occup. Environ. Med..

[25] Baldassarri M., Fallerini C., Cetta F., Ghisalberti M., Bellan C., Furini S., Spiga O., Crispino S., Gotti G., Ariani F. (2018). Omic Approach in Non-Smoker Female with Lung Squamous Cell Carcinoma Pinpoints to Germline Susceptibility and Personalized Medicine. Cancer Res. Treat..

[26] Krabbe J., Ruske N., Kanzler S., Reiss L.K., Ludwig A., Uhlig S., Martin C. (2019). Retrograde Perfusion in Isolated Perfused Mouse Lungs—Feasibility and Effects on Cytokine Levels and Pulmonary Oedema Formation. Basic Clin. Pharmacol. Toxicol..

[27] Engle M.L., Monk J.N., Jania C.M., Martin J.R., Gomez J.C., Dang H., Parker J.S., Doerschuk C.M. (2019). Dynamic Changes in Lung Responses after Single and Repeated Exposures to Cigarette Smoke in Mice. PLoS ONE.

[28] Doz E., Noulin N., Boichot E., Guénon I., Fick L., Le Bert M., Lagente V., Ryffel B., Schnyder B., Quesniaux V.F.J. (2008). Cigarette Smoke-Induced Pulmonary Inflammation Is TLR4/MyD88 and IL-1R1/MyD88 Signaling Dependent. J. Immunol..

[29] Eltom S., Belvisi M.G., Stevenson C.S., Maher S.A., Dubuis E., Fitzgerald K.A., Birrell M.A. (2014). Role of the Inflammasome-Caspase1/11-IL-1/18 Axis in Cigarette Smoke Driven Airway Inflammation: An Insight into the Pathogenesis of COPD. PLoS ONE.

[30] Wong J., Magun B.E., Wood L.J. (2016). Lung Inflammation Caused by Inhaled Toxicants: A Review. Int. J. Chron. Obstruct. Pulmon. Dis..

[31] Ho E., Karimi Galougahi K., Liu C.-C., Bhindi R., Figtree G.A. (2013). Biological Markers of Oxidative Stress: Applications to Cardiovascular Research and Practice. Redox Biol..

[32] Galasso M., Gambino S., Romanelli M.G., Donadelli M., Scupoli M.T. (2021). Browsing the Oldest Antioxidant Enzyme: Catalase and Its Multiple Regulation in Cancer. Free Radic. Biol. Med..

[33] Reisgen M., Thomas K., Beilmann V., Markert A., Gerhards B., Krichel T., Schmidt K., Kraus T., Martin C., Brand P. (2020). Increased Neutrophil Granulocyte and Myeloperoxidase Levels Indicate Acute Inflammation Due to the Exposure of Zinc- and Copper-Containing Welding Fumes. J. Occup. Environ. Med..

[34] Krabbe J., Ruske N., Braunschweig T., Kintsler S., Spillner J.W., Schröder T., Kalverkamp S., Kanzler S., Rieg A.D., Uhlig S. (2018). The Effects of Hydroxyethyl Starch and Gelatine on Pulmonary Cytokine Production and Oedema Formation. Sci. Rep..

