# Supplementary material for: Welding Fume Instillation in Isolated Perfused Mouse Lungs—Effects of Zinc- and Copper-Containing Welding Fumes
Source: Int J Mol Sci. 2022 Aug 12;23(16):9052. doi: 10.3390/ijms23169052 (PMC9408907; doi:10.3390/ijms23169052)
Supplement: Supplementary file 1 [file ijms-23-09052-s001.zip › ijms-1816325-supplementary.pdf]

International Journal of
Molecular Sciences

MDPI

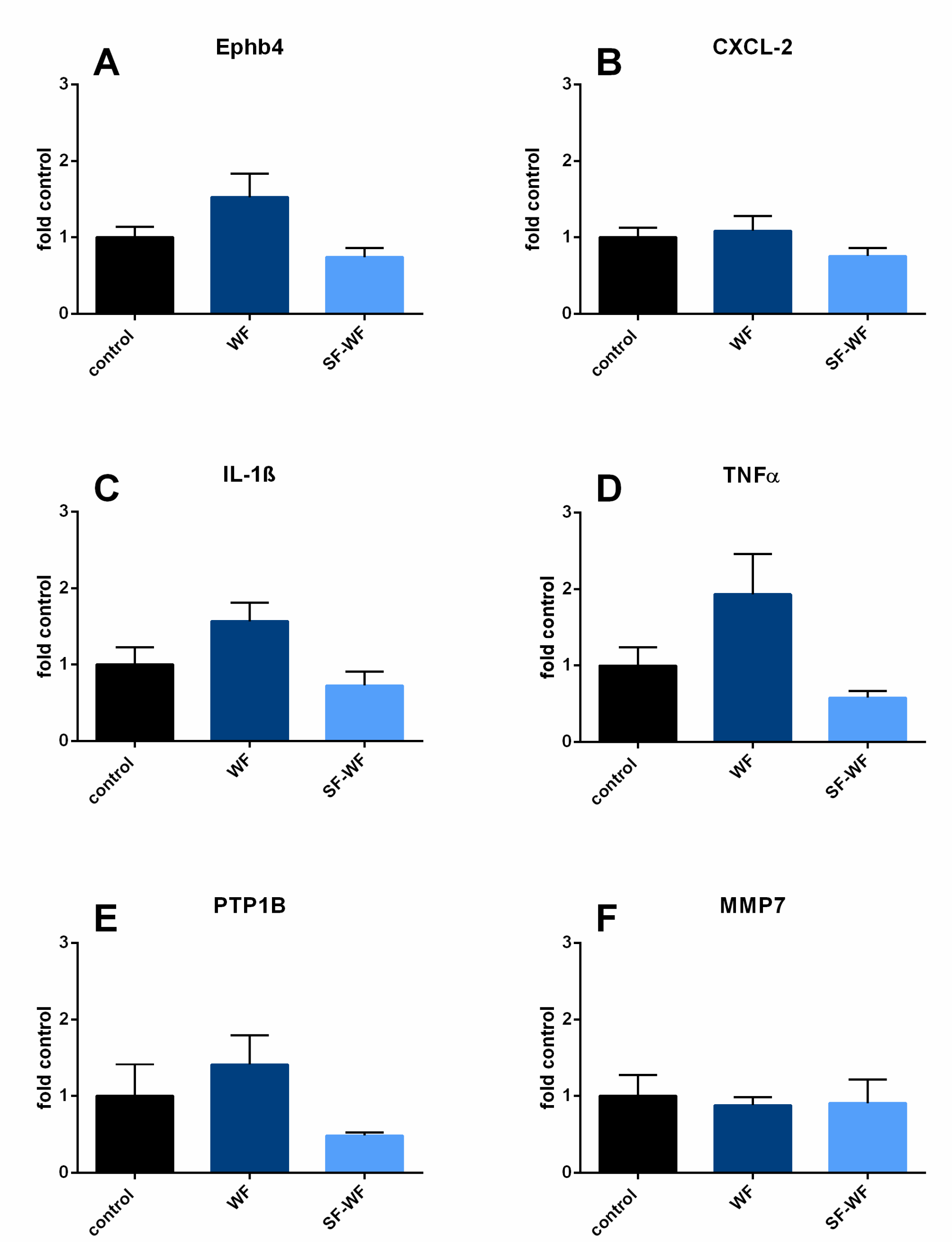

Figure S1. Influence of welding fume instillation: mRNA expression of isolated perfused lungs. (**A**): EphB4 mRNA expression levels (mean ± SEM), (**B**): CXCL-2 mRNA expression levels (mean ± SEM), **C:** IL-1β mRNA expression levels (mean ± SEM). (**D**): TNF*α* mRNA expression levels (mean ± SEM), (**E**): PTP1B mRNA expression levels (mean ± SEM), (**F**): MMP-7 mRNA expression levels (mean ± SEM), control and WF n = 6, SF-WF n = 5.
